# Portrait of an Artist as Collaborator: An Interpretative Phenomenological Analysis of an Artist

**DOI:** 10.3389/fpsyg.2019.00251

**Published:** 2019-02-12

**Authors:** Ian Hocking

**Affiliations:** School of Psychology, Politics and Sociology, Canterbury Christ Church University, Canterbury, United Kingdom

**Keywords:** case study, creativity, collaboration, artist, interpretative phenomenological analysis

## Abstract

The subjective experience of being an artist was examined using interpretative phenomenological analysis (IPA), focusing on the perspective of the artist but interpreted by me, a psychologist, from my perspective as an artistic collaborator. Building upon a literature that has hitherto focused on clinical, elderly, or vulnerable participants, I interpreted superordinate themes of *Process* (Constraint, Playfulness, Movement) and *Identity* (The Ill-Defined Artist, Becoming, Mixing Identities, Choosing an Identity, Calling, Collaboration, and Outsider). These themes are broadly similar to the existing literature, but emphasise identity while de-emphasising self reflection and the need to become an “insider.”

## Portrait of an Artist as Collaborator: an Interpretative Phenomenological Analysis of an Artist

Modern psychology has had a long association with artistic works, examining the psychological characteristics of, for example, architecture (Woelfflin, 1886, as cited by Jarzombek, 2000) and expressionism (Worringer, 1911). With a movement toward the Gestaltist approach (Perls et al., 1951), the field emphasised internal representation, as well as therapy. Interest waned in the 1970s amid criticisms that art itself is too subjective an experience to render using the ostensibly objective framework of psychological theory, and with individual reactions to art being too variable.

The positivist approach, which characterises much of contemporary psychology, argues that observation and experiment are the only sources of substantive knowledge (Colman, 2015). Under this auspice psychology has explored, for instance, aesthetic preference and appreciation (e.g., symmetry and compositional balance; see Lindell and Mueller, 2011). Meanwhile, wider creativity research has explored personality-based, cognitive, contextual, psychometric, psychoanalytic, and pragmatic approaches (Mayer, 1999), but commentators advocate that more dialogue between these areas is needed (Nelson and Rawlings, 2007).

One reason for the separation of quantitative and qualitative streams is the tractability of creative phenomena—broadly defined—at different levels. We can see this separation most clearly in memory research. Our understanding of low-level aspects of memory is well advanced (Baddeley, 2012), but higher-level, and potentially more meaningful research into, say, how memories inform our identity is less coherent, partly hampered by the nature of the phenomenon: it is less suitable to a quantitative, cumulative discipline. In the case of creativity, if the parameters of a creative task are set by experimenters—and thus the motivational and emotional aspects creativity are rendered more artificial—the creative process will be undermined, or at least changed substantially from the process as it manifests in real life. We know, for instance, that individuals have been shown to perform better in problem-construction activities that correspond to their own values and interests (Mace and Ward, 2002). This is an issue, then, of the applicability of much general, quantitative creativity work to those creative individual involved in a particular field of expertise. In recent years, qualitative approaches have grown in popularity (see Smith and Osborn, 2015). These emphasise a deeper, more meaningful analysis of phenomena, and commonly feature a thorough treatment of verbal texts (i.e., any object that can be read).

When we look at research on artists being artists, qualitative approaches predominate, and, among various themes, *identity* in its broadest sense is particularly important. Johnson and Wilson (2005), for instance, studied women who were following a multi-generational discipline of textile handcraft. The study combined questionnaires, historical research and participant observation. Across several meaning-construction themes such as production, and the use of what was produced, identity was overarching; the role of producing the textiles gave them, in some sense, their “place in the world” (p. 118). A similar underlying principle was discovered in a narrative inquiry investigating how unpaid arts and crafts contribute to retired people’s sense of occupational identity (Howie et al., 2004). In this study, where six creative industry participants looked back on their lives, the maintenance of their creative identities was founded upon the social embeddedness of practise, an awareness of themselves and their skills changing over the lifetime, a complementary awareness of certain qualities in themselves being stable, and the opportunity to remain reflective on how their creative products gave them a sense of self, and of their life’s journey. Process—i.e., the sense of identity as a changing, responsive quality—is emphasised in an ethnographic study by feminist author Clark/Keefe (2014). An artist herself, identity for Clark/Keefe is what one *becomes*. Spence and Gwinner (2014) provide a similar narrative on the relationship between art, identity, and mental health by an artist living with mental illness, written in conjunction with an Artist in Residence. One of the important things to come out of this research was the notion of an individual maintaining a duality between their artistic identity and their identity as a person with a mental illness; they are not, therefore distinct, though might be presented as such to the outside world. The notion of being an “outsider” is also important, but in the context of attempting to become an “insider.” This is further emphasised by Perruzza and Kinsella (2010), who reviewed the literature on the usefulness of creative arts occupations for therapeutic practise; they identified several important factors, including collaboration, efficacy, and benefits for individual identity—all implicated, to greater and lesser degrees, in participants’ “sense of self” (p. 265)—as well as their social identity. The importance, again, of identity was reiterated in Reynolds et al. (2011), who studied twelve older female visual artists living with arthritis, finding evidence that their artistic activities helped maintain a positive outlook. Finally, Reynolds and Vivat (2010) examined another sample of older women living with chronic fatigue syndrome (also known as ME); a thematic analysis suggested that the women fell into two groups. For some, their creative works enabled them to recover some of the previous identity that their illness had diminished; for others, their art provided them with a more positive identity, and this group felt that they had become artists. Thus not only is identity central to creative individuals, but *becoming*, or making the transition to artist, can be important too.

Elsewhere, Mace (1997) used a Grounded Theory approach to explore professional artists in New Zealand. They found that *movement*, as a metaphor, was important because each artwork develops over time. The process of artistic creation is viewed as a continuous cycle of problem-finding and problem-solving; communication between these two elements is crucial. For artists, the exploratory stages are sometimes the most engaging, where natural playfulness and freedom add to the enjoyment. Mace and Ward (2002) extended these findings with another Grounded Theory analysis of artists. As before, the emphasis was on a model of artists’ creative process during a time when, importantly, they were producing their own artwork rather than anything specified by researchers. They identified four stages of development: conception, idea development, making the artwork, and finishing. Again they emphasise *movement*, not necessarily linear, sometimes cycling from broad conception to finished artwork. Similarly, physical *constraints*, helpful or not, are critical in the production of art and often shape the nature of the final piece. Another concept suggested is exploration or *playfulness*: being motivated by enjoyment and keeping options open. This stage-like conception of the creative process has antecedents including Wallas (1926), who proposed preparation, incubation, illumination and verification. Preparation involves breaking down the problem and identifying which skills and knowledge will be required to progress with it. Incubation requires setting aside the problem. Illumination is characterised as a sudden insight into the solution, which is then tested during the verification stage. Some consider problem finding to be “pre”-stage (e.g., Amabile, 1996). Others focus on the distinction between implicit and explicit process, which are consistent with two processes: a fast, automatic mechanism and a slow, deliberate one (Allen and Thomas, 2011).

### The Current Study

The above studies are drawn from groups of individuals where responses are pooled by researchers uninvolved in the artistic process itself. Multiple participants can be useful in making conclusions more generalisable, for instance in research on visual artists, for which we know a great deal about the relationship between creativity and perceptual abilities, drawing skills, autobiography and personality (Locher, 2010). This is less informative within a qualitative context; here, we are just as interested in how a given reality is constructed. Furthermore, much of this literature, because it has sought adults spending much of their time in purely artistic endeavours (i.e., in the production of artefacts that are typically novel and valuable), has necessarily tended toward groups of retirees, or those recovering from illness. This is not the approach of the present study. In June of 2015, I was contacted by the organisers of a contemporary art festival who wished to embed an artist within the environment of a researcher examining creativity from a quantitative perspective. This allowed me to study a young professional creative individual over a 12-month period, communicating by way of regular face-to-face meetings, telephone conversations, a shared blog, as well as email; finally, I conducted three interviews investigating themes based on the psychological literature and concepts that appeared to be important from our communications. Crucially, this artistic process was collaborative, allowing me to go beyond the typical “outsider” perspective to an ethnographic or participant observation approach, and addressing the call of Freeman (2014) that artists and psychologists should collaborate in their pursuit of understanding creativity. Locher (2010) observes that our knowledge of the artistic process primarily comes from archival case studies and real-life case studies. The former typically involves the examination of working draughts, such as the those for Picasso’s *Guernica* (see Weisberg, 2004). Clearly, a limitation of this approach is that the work is not captured *in vivo*. Real-life case studies avoid this limitation by analysing the artwork from beginning to end (e.g., Miall and Tchalenko, 2001). Locher goes on to observe that factors related to autobiography, motivation, culture and history will contribute to a final artwork, and the complex interplay between them may be less suited to an experimental approach. Gruber’s evolving systems approach takes a similar stance, where the construction of meaning is emphasised, along with close study of the creator, and consideration of the sociohistorical milieu (Gruber, 1980).

The current study takes the approach of Interpretative Phenomenological Analysis (IPA), a qualitative technique that helps us understand how participants make sense of their personal and social world (Smith and Osborn, 2015). The term “phenomenology” is used in the broad sense of being concerned with subjectivity, rather than the narrower sense in which it is used in phenomenological psychology, which is the application of Continental philosophical phenomenology to academic psychology (Valle et al., 1989). The focus of IPA is a nexus of specific experiences, events and states. It draws heavily upon phenomenology, the philosophical study of consciousness, experience, and the structures that support them. Although phenomenologists do not agree amongst themselves on a formal definition of the term, the current paper takes the approach that phenomenological investigation should be systematic, involve reflection and study, and that the phenomena concerned should be those arising from acts of consciousness. This follows from the work of Husserl (1931) and later thinkers such as Ricoeur (1990) who underscored the complex relationship between meaning, narrative and forms of identity. Nelson and Rawlings (2007) characterise this approach as being more about the “whatness” than the “whyness.” One issue with IPA, which we should bear in mind, is that the descriptions of a person’s internal states and behavioural processes are necessarily limited by their ability to accurately introspect on the processes that generate them (Perkins, 1981, as cited by Mace and Ward, 2002). We should also bear in mind that much artistic work might be intuitive and thus implicit; indeed, for some researchers, this is a hallmark of creativity (Nelson and Rawlings, 2007; cf. Allen and Thomas, 2011).

The construction of meaning is a complex process, and no less so in the context of IPA. We can consider two sources of meaning-making—the individual(s) under study and the person conducting the analysis—but these must be set against the wider complexities of meaning-making in an extra-individual world (Berger and Luckmann, 1991). IPA can be conducted on groups of individuals or a single individual. For instance, in the current study, I, Ian, as a psychologist, will attempt to explore meaning making with the artist, whom I will call Jane; the scope of her experience will be her life in contemporary art, as well as our artistic collaboration. The key aspects of IPA are: (i) an inductive approach, where hypotheses and prior assumptions are avoided; (ii) participants tend to be experts in the area of interest and have the ability to describe their thoughts, commitments and feelings; (iii) researchers reduce experiential data complexity through rigorous and systematic analysis; and (iv) analyses include both an individual, idiographic perspective as well a sense of commonality with other data (Reid et al., 2005). A successful and valid analysis is interpretative (subjective, with no attempt to be “factual”), transparent (where the journey from data to interpretation is clear) and plausible (to the participant, to the researcher, and to general readers). Throughout this process, I bore in mind Mace and Ward (2002) observation: “…the genesis of artwork arises from a complex context of art making, thinking, and ongoing experience” (p. 182).

As there is no prescribed approach for phenomenological methods, it has been argued that it should adapt to the unique qualities of the phenomenon under study (Wertz, 1983). The late stage interviews should be seen in the context of a long term collaboration with the artist; as such, it represents the “tip of the iceberg.” As Tzanidaki and Reynolds (2011) have argued, sample size has traditionally not been seen as an indicator of quality in the qualitative approach because rich data and nuanced analysis often trump quantity. Reid et al. (2005) also warn against assuming a linear relationship between number of participants and the value of research. Further, in the present paper, it is not the intention to present data that has been sampled from a notional population of artists; this is not a strength of the phenomenological approach, even within larger samples, and it is difficult to imagine what the larger population of artists actually would be, given the particularly individual ways in which artists go about their work (and is arguably a central issue for studies where artists of from differing disciplines are mixed, e.g., Nelson and Rawlings, 2007). For this reason, and others, Smith (2004) has argued that there can be advantages for smaller sample sizes and case studies, such as the multiple case studies examining the role of art-making in identity maintenance for those living with cancer (Reynolds and Prior, 2006).

Some personal identifying information has been changed.

## Methods

### Design

This study uses IPA to analyse transcribed interviews conducted with an artist in the 11 month of a 12-month artistic collaboration. As well as these transcripts, analysis was informed by a shared blog, emails, telephone conversations and face-to-face meetings. However, excerpts from the interviews alone are presented here; it was agreed early in the process that making our general communications subject to study would have introduced a harmful self-consciousness to the project.

### Participant and Procedure

The case study involves one artist, pseudonymously called Jane, with whom I worked on a contemporary art installation. The installation involved listening to Jane’s recreations of telephone calls to psychics, with the psychics trying to predict the nature of the installation. The installation took place in a blacked-out hut.

We spoke three times over 2 weeks for a total interview time of 3 h 30 min. A small amount of the transcription was done by a student intern and myself, but the majority by a graduate student. The final text base was 28,000 words.

Given the importance of “bracketing” presuppositions in IPA, the author underwent an initial self-reflective process that focused on the artistic collaboration from their perspective, their own artistic endeavours (in this case, novel writing) and the creativity literature (cf. a similar approach taken by Nelson and Rawlings, 2007). Additionally, throughout the artistic collaboration and during data collection, a reflective diary was used to assist in the process of reflection on the interviewer’s thoughts and feelings (cf. Savin-Baden and Fisher, 2002); this was not to eliminate bias, which is inevitable, but acknowledge the presence of the researcher in the research process, helping to identify themes, and helping to enhance the research process (Finlay and Gough, 2008). This reflection brought home several points, which are personal to me and, whether or not they are factually correct, describe my views: I feel that art and artists are crucial to a functioning society, given the human need for expression and the value placed on the products of these expressions; my quantitative approach is perceived by most artists as reductionist; as a published novelist, I have some common ground with artists; openness on my part was crucial in the collaboration; the typical psychologist-participant power dynamic needed to be minimised but acknowledged.

To guide the interviews, I used a semi-structured format based on core, open categories: history, which focused on personal biography; views, which focused on what art, creativity and practise meant to the artist; and collaboration, which addressed previous collaborations as well as the present one. I made sure to touch upon the following concepts: work/life balance, identity, nature of creativity, collaboration, quantification, documentation, narrative, privacy, power/authority, prediction, and flow. I would introduce these by saying, for instance, “Now, I want to talk about identity. How would you describe your identity?” Or I might say, “Tell me about the role that work/life balance plays in your art.” As we spoke, I made notes to record my thoughts, help think of further questions, and to guide my later interpretation of the interview transcript. The transcript was then read carefully and annotated with notes on particular meanings, which were then collected into the themes below.

While this paper examines individual components of experience, it does not present experience as a separate entity alongside other concepts; I see it as a higher level construct that draws upon all concepts. Together, these comprise my interpretation of Jane’s experience.

The study received ethical clearance from the Research Governance Committee of Canterbury Christ Church University (Ref: 16/SAS/277C). The case study was conducted with the full informed consent of the artist. This was signed prior to the interviews. She has also viewed and approved this final version of the article.

## Results and Discussion

Jane has read this manuscript. I have maintained the broad direction of my interpretation, but some of her comments are included as footnotes.

Jane is a professional artist in her mid-thirties. She started out as a painter but soon became interested in more contemporary forms of expression. For her, a key transition point was saving enough money to attend a “studio” programme abroad, after which she worked on what she now considers to be an artistic performance of the type she now pursues.

That’s the first [artistic] work. That marks the point where I thought “I’ve found something interesting that isn’t just drawing or dealing with something in a slightly…” Looking back, that feels like the work that marked the start of being an artist. (396)

## Superordinate Category: Process

### Subordinate Category: Constraint

Jane appears to view orthodoxy as something that can be pushed against, tested, or broken. She sees orthodoxy as arbitrary and sometimes limiting. Challenging orthodoxy can be seen in some of her works, such as an installation that involved her wearing all her clothes at the same time. On the face of it, this is absurd, but can make the audience wonder why a particular way of dressing should be absurd, and what this might say about consumerism.

I suppose there’s that, sometimes I want to respond to things, just like the idea of going crazy or doing something stupid, kind of breeching those “norms” which comes back to that normative ways of doing things. (882)

This corresponds with the artist as an explorer who isn’t necessarily concerned, from the outset, where they might end up, which Nelson and Rawlings (2007) characterise as an attitude of risk-taking, of “engagement in a process of exploration without knowing exactly what is being looked for” (p. 222).

When she worked for her previous employer, Jane didn’t like the constraints imposed by the system surrounding the job, particularly having to move in a direction that wasn’t entirely consistent with her political position.

I found it very constraining and now I really enjoy what I do, and I don’t quite know what that says about me. (1359)

This is not necessarily something unique to Jane, but it forms an important part of her identity. Reaction against constraint has long been considered an important quality of successful artists (though not for those where artistry is seen more in terms of a trade, e.g., the pre-Romantics; Brown, 1991). Shulman (1984), for instance, discussed the nineteenth century writers Hawthorne, Melville and Poe in context of their metaphorical prisons, where the prison is formed from artistic heritage: like prisoners, they feel a sense of enclosure; they work out ways of defying authority; they attempt to communicate with those on the outside. This is taken further, of course, with Postmodernism, where there is arguably even greater reaction against constraint, particularly those associated with Enlightenment rationality (Butler, 2002).

### Subordinate Category: Playfulness

Closely related to this constraint—cf. Jane’s use of “enjoy” in the above quote (1359)— is playfulness. This, for Jane, is about taking the everyday (and occasionally the less obvious) and giving herself the freedom to play with it, much as a child might play with a cup or a word. Convention-breaking features prominently in this, as does repurposing; putting something to a use that strains against the intention of the creator, or at least the normative use.

So what are the systems at work? What are the conventional ways in which things are being done? How I can use my practise to kind of intervene and who will understand that? Maybe play with it, and transform it, or subvert it somehow. (218)

In the above quote, Jane gives two elaborations of her “play” concept. The first is “transformation.” This seems to hark back to an important tenet of what creativity means for most people: that is, creativity takes the raw materials of skill and experience to produce something new in the sense of being recombined or mixed. This clearly important for Jane. The artist is a lens between her audience and what she sees. The second term is “subvert”: to undermine, destabilise, or unsettle. Jane seems to be using the term here in the broader sense of repurposing something for a use that is not intended. This, of course, is a shortcut to defamiliarisation, which allows the audience and the artist to go beyond the superficial, everyday conception of thing to a deeper understanding, or at least a reaquaintance with its nature. This reminds us of the classic Alternative Uses Task (Guilford, 1967), where participants must come up with different ways to use everyday objects, such as a brick, paperclip or newspaper. Alternatives to everyday or mundane function relates to avoiding cliché in fine art and fiction: a cliché like ‘it was a dark and stormy night’ is so common it will be hardly read; subverting the phrase to something like “It was neither dark, nor stormy, but night all the same” will cause the reader to reengage, and perhaps consider cliché in general.

The playfulness is an important part of collaboration, too. It’s related to testing and trying out ideas.

Any opportunity to work collaboratively, that sort of playfulness, reels me in, it’s fun and awful [Jane laughs], definitely interesting. (784)

This use of playfulness is subtly different. It’s playing in the sense of bouncing ideas off people, of being surprised by them, and allowing a collaborator to introduce the unexpected, in a kind of third-party incubation (Wallas, 1926). This ties in with studies of visual artists, which show general agreement that such artists have no final image in mind before they start to sketch or paint (see Locher, 2010). There is an emotional, fun component to Jane’s playfulness. Again:

…working on lots of field recordings, and I did some documenting. That was the first time that I collaborated and it was just really fun. (1004)

The playfulness also ties in with representation. Jane is clear that what she does as an artist goes beyond what might, at first blush, be termed simple representation. A photograph, for instance, is a relatively faithful representation of the physical characteristics of something external to the camera on that occasion, skewed somewhat by the camera’s physical properties, post production, as well as the choice of the photographer to capture and present that particular moment. A photograph is not truly a simple representation, but it is comparatively simpler than the kind of contemporary art that features in Jane’s portfolio. She talks about this in the quote below.

So, what’s the difference between an anthropologist and an everyday artist, and the conclusion I came up with, was that it’s something to do with representation, whereas an artist you can be very very playful, and your goal isn’t necessarily to represent, or my goal is not to represent, it might be more to play with misrepresentation or sort of like tease, play more of a trickster role in a sense. So, it’s not always really serious. …It’s saying that I don’t really agree with that, and although some of the methods might be similar in just participating in the situation, being a participant, by making something, or turning something into some kind of knowledge, there is more scope to be playful or misrepresentative. (826)

Playfulness can be seen as a delay of closure; the idea that one is putting off the serious work of completion, after which there is no further opportunity for play. Getzels and Csikszentmihalyi’s (1976) longitudinal study of artists suggested that artistic success was related to “delay in closure,” that is, putting off the inevitable moment when an artist must commit. This point is reiterated by Mace (1997), who states that the artists in her study had an excitement with, and preference for, the experimental stage of the artistic process. This might be related to a lack of concern with goal-focused behaviour, or a wish to dwell within the part of the process that is most flexible and unset. There is also a sense in which it is hard to identify when an artwork is finished, perhaps due to a difficulty in objectively evaluating the artwork while still retaining an emotional connexion to it (Mace and Ward, 2002, p. 191). This also touches upon what (Nelson and Rawlings, 2007) have called the “freedom-constraint dynamic,” which is about having enough freedom to be creative, but not too much—as mentioned earlier in this paper, constraints are important in providing a path, even if they turn out to be not directly important in their own terms, like a “soup stone.”

### Subordinate Category: Movement

Jane often spoke about the role of movement, which can be metaphorical as well as literal. It is linked to coming at something with fresh eyes but goes beyond newness for its own sake. Context is important in her work and, with changing context, comes changing ideas. In his interviews with older American artists, Santlofer (1993) makes the point that these artists are always on the look-out for discovery, including self-discovery: “constant struggle and reevaluation [is] inherent in the creative process” (p. 87).

Jane is interested in exploring, so novelty^1^ is an important aspect of her work. She says, for instance,

I kind of feel that there is a role in being able to move around and I suppose, to offer a different perspective—questioning. This kind of “questioning” function—a challenge—a question, which is definitely a challenge function. (618)

This attempt to see things from a different angle, and distance, is similar to the theme “distant-engagement” identified by Nelson and Rawlings (2007) as “an alternation between immersion in the manipulation of material and distancing oneself” (p. 221).

Here is an example of movement in a metaphorical sense, connected to Jane’s common practise of creating works that are very much “new”^2^ in the sense that, for her, she is not repeating herself:

I don’t like remaking, previous work—or restating previous work. I always like doing something different and moving on. (1413)

Though the movement often involves geographical travel, I felt that movement as a metaphor for travel and change is most attractive to Jane. When I asked her outright about the importance of travel, she was quick to identify its limits:

I don’t think it needs to be travelling to somewhere new. I don’t think it needs to be that at all. I think, often, through having done lots of residencies, for example, and having produced work through that, you are somewhere different. (201)

Turning to an artwork that explored the concept of risk assessment, she goes on to say:

I was in an art school and I just became very interested in this kind of form of procedure. (201)

So movement can be an artistic driver for Jane, but the connexion to movement is not a simple one. It involves the notion of “edge walking,” or the artist being an outsider making discoveries with fresh eyes, and producing an artwork that sparks off this unfamiliarity^3^. The idea that unfamiliarity is consistent with examining more closely is echoed in Freeman (2014), who looked at artists drawing inverted faces, which are not organised according to well-known principles that shape the drawing of upright faces. Freeman goes on to write that trained artists gain familiarity with both their medium and their subject; this can lead to a kind of abstraction, or overview perspective, where the structure of the whole subject becomes as important as the details found within the structure. The movement can also be a form of escapism in its non-perjorative sense; Fisher and Specht’s (2000) study of older artists called this “escaping the mundanity of life,” as well as its aches and pains. There is an obvious connexion to Csikszentmihalyi’s (1997) concept of flow, and recent work by Zimbardo and Boyd (2008) on time perception: for them, individuals involved in creative endeavours (particularly when it provides immediate feedback) are likely to be more present-focused and hedonistic.

## Superordinate Category: Identity

### Subordinate Category: The Ill-Defined Artist

Her identity as an artist is something that Jane has thought about and perhaps struggled with, though I think I might have been making more of this than she did; I was particularly interested how she saw herself, both as an artist and creatively.

I remember the moment when you think, “Am I an artist? Am I not an artist?” What do you have to be doing to be an artist? Do you just have to, like, say I’m an artist? What qualifies you to be an artist? It’s quite interesting. (458)

Later in this response, Jane talks about the artistic identity as being related to what is *done*. That is, action is a crucial component. One is, therefore, an artist because one attempts at art. This contrasts with the position of Clark/Keefe (2014), in which an artist is something that ones becomes, rather than is or is not.

For Jane, art does not have to take up a majority of one’s time.

Lots of artists, because of the fact that you can’t make much money through being an artist […] you have to find different jobs. You might work in construction, you might do teaching, and do all sorts of things. (458)

This comes back to a method of going about things. The artistic identity is one of method.

So, for me, my view on my art is kind of a way finding some sort of liminal way of operating. (882)

This touches upon a point made by Fisher and Specht (2000), who studied older artists. They seemed to have their identify as artists shored up by their sense of self in terms of competency and efficacy, what Herzog and House (1991) have referred to as the “agent self.” For Jane, there seems to be a sense in which the performance of art is “liminal,” operating on the threshold of art and not-art, or the familiar and unfamiliar.

### Subordinate Category: Becoming

For Jane, making the transition between a less fulfilling professional career to the more interesting, but risky, career of artist was, obviously, important. She told me that she was interested in art from a young age, but was influenced to follow an academic career. At school, she had an art scholarship, and she considers art one of her best subjects. In terms of “becoming,” this, presumably, is the same struggle that afflicts all those who make the transition from the more orthodox, salaried track to the arts sector. Throughout the interview, I got the sense that she saw her life—her professional life, at least—as dividing very much into two. Indeed, when she first became active in the artistic community as a practitioner, she was not keen to disclose her former profession. This aspect chimes with the position of Clark/Keefe (2014); an artist is something one becomes.

And to start with, when I became an artist, I just didn’t talk about that period of my life at all. I didn’t want to identify with it, didn’t want to bring it up, because how can those two things… Those things feels diametrically opposed, like the [previous employer] and the artist. (466)

This sense of becoming is linked to expertise. This might be a categorical distinction for a third party observer, but for the artist the concept is more nuanced. An artistic expert, after all, is no more than a person with more mature creative processes (Mace and Ward, 2002). Additionally, artists with experience are more likely to know what can lead to success and failure. The kind of knowledge built up, according to Mace and Ward (2002), is “explicit and implicit understanding of techniques, skills, art genre, art theory, aesthetics, emotion, values, personal theories, personal interests and experience, previous work, and historical and contemporary art knowledge” (p. 183).

### Subordinate Category: Mixing Identities

Immediately after saying the above about the separation between two professional identities, she adds:

But not necessarily. (466)

This reflects her belief that two identities might have appeared separate at the time, but from her present day perspective, they are less far apart. The separation seems less obvious given her experience now of being an artist.

### Subordinate Category: Choosing an Identity

For Jane, one difference between being in her previous employment and being an artist seems related to self control. As an artist, you are, for better or for worse, your own person. Whereas, her previous employment had characteristics that led to situation where…

…you have to sacrifice your own identity, really. Completely. You have to… Yes, you’re doing a lot of problem solving, but you have to conform to the system, and I don’t like conforming to systems, have never. (482)

For Jane, then, this sense of ownership, or personal sovereignty, is an important part of being an artist. It also fits with what she sees as her non-conformist, iconoclastic attitude.

### Subordinate Category: Calling

There is a sense in which the artistic identity is all-encompassing. Because it runs like a thread through everything, Jane rarely “switches off.” This is exacerbated by the sporadic nature of the freelance work, as well as its intrinsically enjoyable nature (see *Playfulness*), and “flow.”

It’s not your job, it’s a whole identity… you don’t quite know when the next pay cheque will come up, so you end up actually, well I do anyway, having a lot on and not eating until nine o’ clock or eleven o’ clock (642)

The idea of calling has been linked to an individual’s search for meaning in life and, for some researchers, this search for meaning is our primary drive (Frankl, 1985). Dobrow (2013) followed musicians over 7 years to identify factors related to their calling. She found that, far from being a stable construct, calling changes over time, which is consistent with Jane’s change from a person who is interested in art to a person who actively produces art and is part of the local and wider artistic community.

### Subordinate Category: Collaboration

From the perspective of collaboration, Jane sees her identity as changeable and responsive to context. Collaboration also raises the issue of authorship; during collaboration, there is sense in which authorship is challenged. In the quote below, Jane refers to a previous collaboration in which an artistic colleague was offered, and used, Jane’s hard drive in an art installation. The collaboration raised issues of control and boundaries.

I’d like to think that authorship – I’m very lazy [Jane laughs] about it. But it’s interesting because that experiment [involving the handover of the hard drive] proved that there are certain things, which I feel a part of my identity as an artist, which is sort of the way in which I do things and it felt uncomfortable having someone replicate that so precisely. (1072)

The use of “lazy” above is interesting; it seems to be more about being patient and able to delegate, both of which are parts of her strategy to avoiding repeating herself in art. Indeed, collaboration, far from being an unusual part of the creative process, is fundamental to Jane.

I am producing some knowledge about something which takes a form of art but often it involves engaging with other people, so I think its more about that engagement with others and how do you represent that in the artistic process. (132)

And:

I tend to just find it really interesting listening to other people, I have to say. (164)

There are downsides to this collaboration. In our collaboration, she sometimes felt she was being measured and judged by me. Here is an excerpt including us both:

Interviewer: So [the middle of the collaboration] was that a kind of … that was an anxious time. (1680)

Jane: Yeah. Because I think, I initially had this sort of anxiety around [the question], “Would I be negatively impacted from through just being a participant?” And through having my process observed and particularly I was worried about [our private, collaborative blog] because…and […] like when I was putting this blog post up today…I was like, “Do I really want to post that? Do I want to send that?”

### Outsider

The concept of the outsider was raised repeatedly throughout the interview, mostly by me, reflecting my own notion of what the “typical” artist does. Jane is well aware of the literature on the role of a certain type of artist, that the idea of what she calls an “edge walker” is connected to the notion that she, as an artist, works best on the periphery or borderline. In this view, the artist is a person who takes a different perspective for a viewpoint advantage, just as a person walking a ridge can see down both sides of it^4^. Jane can be physically outside, or displaced, too.

I always end up immersing myself in different situations where I’m quite…I don’t know much about psychology, but here I am, so I’m like, “Oh that sounds interesting, I’d love to do that.” (132)

Jane goes on to say that this movement to the “outer” or “outside” realm is an important part of the fluidity of the creative process, which connects to my own experience of the artwork changing over the 9 months of our collaboration.

I…often [feel] like an outsider in different places from having moved around a lot, and not having a sense of, well, this is my home, this is my culture, but seeing that there is something more fluid. (164)

The notion of being outside feeds somewhat into her identity of *becoming*; because she didn’t become a professional artist as early as some of her artist friends, this helps avoid what she terms an “artist” bubble.

And I think that because I’ve moved around a lot, been heavily involved in lots of different professional different systems … I’m interested in the fact that things operate really differently outside to the art world, than inside the art world. So, I think you can get a bit of a—I’m making huge generalisations here [Jane laughs]—that you can get into a lot of art bubble I suppose, if you go through art school, all your friends are artists who are going to art school, you carry on working, or you know, a lot of my friends aren’t artists, they’re not in that bubble, they struggle to make sense of contemporary art. So, yeah, it is sort of a different perspective on things. I have friends who are bankers.

## Concluding Comments

The present study looked at the way in which one contemporary artist sees herself and her work, taken from the perspective of her collaborator.

The model outlined above—Process and Identity—suggests a separation between the components, but they are, unsurprisingly, well connected. Process, with its ideas of *constraint*, *playfulness*, and *movement* in all forms, are in many ways a reflection of Identity. Here, I’ve broken down identity into several elements, the first of which is the *ill-defined artist*: what we mean by art, artist, and creativity are questions that Jane touched upon throughout her interviews and our collaboration. There is a sense in which keeping these ill-defined allows for a protean, shifting and flexible self-characterisation that keeps avenues of expression open. The second, closely related concept is *becoming*: I use this in the sense of making the transition from the amateur to the professional, or in, another sense, reaching the point where Jane felt comfortable self-identifying as an artist. It involves expertise, commitment, and sacrifice. Third is *mixing identities*: Jane does not see her life as divided into sections where she is totally one thing or the other; as well as being an artist, she is a mother, friend, academic, and so on. *Choosing an identity*, the fourth strand, is about taking ownership of one’s identity, particularly when pitched against jobs or situations (such as her previous employer) in which conforming to a system can involve a “sacrifice [of] your own identity” (Jane: 482). For Jane, an important part of being an artist is regaining, and maintaining, sovereignty over one’s identity. *Calling*, the fifth strand, emphasises the all-encompassing nature of being an artist; meals, and much else, might be skipped in the service of art. This art-first approach was evident during our collaboration, but, as suggested by Dobrow (2013), the vocational sense is likely to change over time. Certainly, it would have been strong at the point Jane chose to pursue a path that took her away from a well-paid job with clear progression. The sixth part is *collaboration*, which brings with it issues of authorship and ownership; these can sometimes overshadow the art, but Jane sees the art that she produces as essentially collaborative, particularly in understanding the potential of the final artwork from the perspective of her “official” collaborator—me—and others (such as, in the case of our artwork, telephone psychics). Lastly, there is the concept of the *outsider*; Jane was wary of facile perceptions of the artist as an outsider. For her, this was rather more staying outside the “art bubble” (Jane: 585) than taking an “objective” stance toward her art. There is a sense in which being within this “art bubble” can lead to a parochial or less interesting approach.

Thus, at the end of this process, and though a psychologist/artist collaboration of the kind called for by Freeman (2014), I was able to identify superordinate themes of *Process* (Constraint, Playfulness, Movement) and *Identity* (The Ill-Defined Artist, Becoming, Mixing Identities, Choosing an Identity, Calling, Collaboration and Outsider). These are broadly similar to themes found in previous research cited in the Introduction and throughout this paper, which often draws from clinical, older, vulnerable or otherwise special participants, suggesting a commonality between these and the professional artist described here, though some qualities, such as self reflection (cf. Howie et al., 2004), the need to become an “insider” (Spence and Gwinner, 2014) were less important for Jane.

## Author Contributions

The author confirms being the sole contributor of this work and has approved it for publication.

## Conflict of Interest Statement

The author declares that the research was conducted in the absence of any commercial or financial relationships that could be construed as a potential conflict of interest.

## Footnotes

1 Jane writes: “Not novelty. I’m not actively in search of the “new.” It’s more that I end up in situations I’m not an expert on, so I often assume the role of the novice, and end up having to familiarise myself with new situations.”

2 Jane writes: “Though of course new works will build on previous works. The ideas I’m interested in don’t change.”

3 Jane writes: “I think this is more about the artist adopting different roles or positions to explore how knowledge is constructed. I think “the artist being an outsider making discoveries with fresh eyes” sounds a bit cliched. It’s not that simple. Being an outsider making claims on behalf of others is something I would never want to be associated with doing. Thinking critically about what it means to be an outsider, yes. Exploring power and positionality, yes. Juxtaposing different ways of knowing and doing, yes. Exploring the feeling of unfamillairity that comes with being in a new situation, and using this as a trigger for work, yes. I’d just be very wary of ever claiming to have fresh or privileged eyes.”

4 Jane writes: “I’m just interested in an artistic practise at the juncture of social encounters, and what happens when different world views come together.”
